# “You’re stuck in the middle here”: a qualitative study of GPs’ experiences of managing knee pain attributed to a degenerative meniscal tear

**DOI:** 10.1186/s12875-023-02075-9

**Published:** 2023-06-21

**Authors:** Helen O’Leary, Katie Robinson, Liam Glynn, Brian Lenehan, Karen McCreesh

**Affiliations:** 1 School of Allied Health and Health Research Institute, Faculty of Education and Health Science, University of Limerick, Limerick, Ireland; 2 School of Allied Health and Ageing Research Centre, Health Research Institute, Faculty of Education and Health Science, University of Limerick, Limerick, Ireland; 3 Graduate Entry Medical School and Health Research Institute, University of Limerick, Limerick, Ireland; 4 Chief Clinical Director UL Hospitals Group and Consultant Orthopaedic Surgeon, Orthopaedics and Trauma Department, University Hospital Limerick, Limerick, Ireland; 5 School of Allied Health, and Ageing Research Centre, Health Research Institute, Faculty of Education and Health Science, University of Limerick, Limerick, Ireland

**Keywords:** Degenerative meniscal tear, Qualitative research, Attitude of health personnel, Family practice

## Abstract

**Background:**

Exercise is the recommended first-line therapy for a degenerative meniscal tear (DMT). Despite this, knee pain attributed to DMTs are a common presentation to specialist orthopaedic clinics. In the primary care setting, the general practitioner (GP) plays a central role in managing patients with knee pain, but to date their perspective has not been explored in relation to DMTs. This study explored GPs’ experiences of managing people with knee pain attributed to a DMT.

**Methods:**

A qualitative research design was adopted and practices in the South and Mid-West of Ireland were contacted via recruitment emails circulated through professional and research networks. Interested GPs contacted the researchers via email, and purposive and snowball sampling was used for recruitment. Semi-structured interviews were conducted online or over the telephone. Interviews were digitally recorded and transcribed. Data was analysed using an inductive approach to thematic analysis. Ethical approval was granted by the Irish College of General Practitioners (ICGP_REC_21_0031).

**Results:**

Seventeen semi-structured one-on-one interviews were conducted. Three main themes were identified with related subthemes: (1) GPs’ experiences of relational aspects of care, (2) GP beliefs about what constitutes best care for patients with a DMT, and (3) how GP practice is enacted within the current healthcare setting. GPs described the challenge of maintaining a strong clinical alliance, while managing perceived patient expectations of a ‘quick fix’ and advanced imaging. They reported slowing down clinical decisions and feeling ‘stuck’ with limited options when conservative treatment had failed. GPs believed that exercise should be the core treatment for DMTs and emphasised engaging patients in an active approach to recovery. Some GPs believed arthroscopy had a role in circumstances where patients didn’t improve with physiotherapy. Limited access to public physiotherapy and orthopaedic services hampered GPs’ management plans and negatively impacted patient outcomes.

**Conclusions:**

GP beliefs around what constitutes best care for a DMT generally aligned with the evidence base. Nonetheless, there was sometimes tension between these beliefs and the patient’s own treatment expectations. The ability to enact their beliefs was hampered by limited access to conservative management options, sometimes leading to early escalation of care.

## Introduction

Musculoskeletal presentations represent a significant proportion (20%) of general practitioner (GP) workload [1]. In the UK, knee pain is the most common musculoskeletal presentation in general practice, after lower back pain [1]. The burden of knee pain on healthcare resources is increasing, and knee pain accounts for the greatest number of new consultations seen in Irish secondary care orthopaedic clinics with meniscal pathology being the most common knee diagnosis after osteoarthritis [2, 3]. Meniscal pathology in adults who are middle aged (35 years) and older, is frequently referred to as a degenerative meniscal tear (DMT) [4], and is considered to be an early part of the whole continuum of degenerative knee disease [5]. A DMT is different from an acute meniscal injury associated with significant trauma or sports injury. DMTs are usually considered atraumatic and often present spontaneously or after low-energy injuries [6].

Exercise therapy, as part of a conservative management approach, is recommended as first line treatment for a DMT [7]. Meta-analyses of clinical trials demonstrate that improvements in pain and function with exercise therapy are comparable in effect size to improvements with surgical treatment, over three-to-60-month follow-up studies [8]. Based on these trials, clinical practice recommendations advise against the use of meniscal surgery in favour of a conservative management approaches such as exercise therapy, oral or topical pain medication or injections [7]. Nonetheless, while the rate of surgery (i.e. arthroscopic meniscectomy) globally has declined over the past two decades [9–12], it remains a commonly performed orthopaedic procedure. In England, the annual incidence of surgeries was 120 per 100,000 persons in 2017 [13]. A similar but higher annual incidence was reported from the Netherlands in 2014 (164 surgeries per 100,000 Dutch inhabitants) [10] and from Florida, USA (291 surgeries per 100,000 persons) in a 2018 study [11].

Qualitative research with people attending Irish secondary care with a diagnosis of DMT identified an expectation of surgery and a reluctance to engage with physical activity for fear of exacerbating structural damage [14]. The beliefs of primary care health professionals, including GPs, were identified as contributing to these negative expectations in relation to the role of exercise therapy in managing DMTs [14]. The influence of clinicians’ beliefs on a patients’ understanding of their musculoskeletal problem and their recovery expectations is well established [15].

Achieving implementation of clinical practice recommendations can be challenging. Research in low back pain suggests GPs have substantial awareness of guidelines, but the practical implementation is more uneven. Providing patient centred care within a broader psycho-social context and operating within a limited health service are some of the challenges to applying low back pain guidelines that GPs face [16, 17]. Numerous studies have also explored the barriers to implementing clinical guidelines for osteoarthritis in primary care [18], but none specifically exploring practice related to DMTs. While there is overlap with osteoarthritis management there are also distinctions, for example, there exists a surgical treatment option for a DMT that is no longer recommended but has not been fully de-implemented [19]. Exploring GPs’ experiences of working with individual patients with DMTs and understanding the wider contextual factors at play is warranted to further explore gaps in the implementation of clinical practice recommendations. Hence, this study aimed to explore GPs’ views and experiences of managing people with knee pain attributed to a DMT.

## Methods

### Study design

A qualitative research design was adopted and the Consolidated Criteria for Reporting Qualitative Studies (COREQ): 32- item checklist was used to guide the conducting and reporting of this study [20]. Ethical approval was granted by the Irish College of General Practitioners Research Ethics Committee (ICGP_REC_21_0031).

### Setting, participants and recruitment

GPs with experience of working with adults with knee pain were recruited from practices in the South and Mid-West of Ireland via recruitment emails circulated through professional and research networks. Interested GPs contacted the research team via email. Snowball sampling was employed as recruited GPs were asked to forward study details to colleagues and purposive sampling was used in the later stages of recruitment to capture variation in demographic factors or key characteristics that could influence GP perspectives. These characteristics related to the practice (location and approximate proportion of patients who had private health insurance) and to individual GPs (gender and years of experience).

### Data collection

Semi-structured interviews were used to explore the experiences and views of the GPs on the research topic. Due to covid-19 related public health restrictions in place at the time of data collection, interviews were conducted online or over the telephone, between September 2021 and February 2022. An interview guide was developed **(**Table 1**)**, informed by the evidence base and clinical experience of the research group, piloted, and revised before study commencement. The interview guide contained six main questions exploring GPs’ practices when assessing and managing degenerative meniscal tears, and factors influencing and informing their decision making. Questions explored particular challenges encountered when managing this type of knee pain and sought suggestions about what could improve practice.

All participants provided informed consent via email, and this was confirmed verbally prior to interview. The interviewer (HOL) was a post-doctoral researcher with experience in qualitative research, who was also a practising musculoskeletal physiotherapist. Participants were aware of the interviewer’s clinical role. Recruitment and data collection continued until data saturation (the point where no new themes emerged from the data analysis) was achieved [21]. An additional two interviews were carried out after this point. Field notes were recorded during and after interviews. Demographics of the participants including age, gender, years of GP experience, and characteristics of the medical practice were collected at the end of the interview. All interviews were video, or audio recorded, digitally transcribed, and checked for accuracy by the researcher. Personal information was removed, and participants were assigned pseudonyms for analysis.

**Table 1:** Interview vignette and guide

Vignette: The following description was provided to GPs at the start of interview: “*The type of patient with knee pain I’m interested in discussing is a middle-aged (in their late 40’s or 50’s) person, who consults with knee pain, which is ongoing for a few weeks, aggravated by weight bearing activities and importantly they have no history of significant trauma. Pain is localised to the medial aspect of the joint and they may also report some accompanying clicking and intermittent locking sensations. From your history/exam you are not thinking this person has osteoarthritis but considering meniscal pathology or a ‘cartilage’ problem. Different approaches can be taken with this type of presentation, but I am interested in your insights and your experiences in helping patients with this type of knee complaint.”*	
1. Can you tell me about your approach to assessment here and any key pieces of information you are looking for? 2. Can you describe your approach to managing patients with this type of knee pain? 3. Can you talk to me about what informs your management of patients with degenerative meniscal tears or early degenerative changes? 4. Could you describe any onward referral and any factors influencing your decision making around this patient? 5. Can you describe any particular challenges you’ve encountered when managing these patients? 6. What if anything would make your job easier when caring for these patients?	

### Data analysis

Transcripts were imported into NVivo software (version 12) and analysed using an inductive approach to thematic analysis, guided by the research aim and Braun and Clarke’s six-step approach to thematic analysis [22]. Using an iterative process, two researchers (HOL & KR) independently coded a sample of transcripts on a line-by-line basis, coming together to review and reconcile coding decisions. Researchers (HOL, KR, KMC) met regularly to review ongoing analysis, examine overlapping codes and patterns in the data and identify emerging themes. In the final stage, potential themes were reviewed and discussed with all research team members, who provided expert insight on general practice, musculoskeletal disorders, orthopaedics and qualitative research methods. Member checking was carried out whereby interpreted data was emailed to a sub-set of participants (n = 3) who evaluated the accuracy of the interpretation and provided feedback.

Strategies adopted to enhance the rigor of the study and ensure the trustworthiness of the research included; debriefings with a team member (KMC) to discuss completeness of data and identifying new areas to explore in subsequent interviews, involving two team members in independently coding data, data saturation confirmed in the analysis, member checking of the analysed data, presenting information about participant characteristics in the results, and supporting findings with direct participant quotes. Reflexivity practices employed during the study included expressly reassuring participants during interview that all views and experiences of services and treatments for this population were of interest. The researcher also considered potential influences of her physiotherapy background on data analysis and write up especially related to views on exercise and conservative interventions. This explicit awareness was discussed among the research team during analysis. Articulating her perspectives to the research team was part of the continual reflexive process to foster awareness of her underlying perspectives and assumptions when engaging with participants and the data throughout the research process [23].

## Results

Seventeen GPs participated in interviews lasting between 35 and 62 min (mean 46 min), 15 were online and two were by telephone. The characteristics of the sample are presented in Table 2.

**Table 2:** Characteristics of GPs interviewed (n = 17)

	Gender	Years in practice	GP position	Practice Location
GP1	Female	9	Salaried	Semi-rural
GP2	Male	11	Salaried	Semi-rural
GP3	Male	36	Locum	Semi-rural
GP4	Male	33	Salaried	Semi-rural
GP5	Male	24	Partner	Urban
GP6	Male	35	Partner	Rural
GP7	Female	20	Salaried	Rural
GP8	Male	40	Locum	Rural
GP9	Female	3	Salaried	Urban
GP10	Male	6	Salaried	Urban
GP11	Male	30	Partner	Semi-rural
GP12	Female	6	Salaried	Rural
GP 13	Female	12	Partner	Urban
GP 14	Male	7	Salaried	Urban
GP 16	Male	37	Partner	Rural
GP 15	Female	10	Salaried	Urban
GP 17	Female	26	Partner	Semi-rural

Data were categorised into three themes with related subthemes: (1) GPs’ experiences of relational aspects of care, (2) GP beliefs about what constitutes best care for patients with a DMT, and (3) how GP practice is enacted within the current Irish healthcare setting.

## Theme 1: GPs’ experiences of relational aspects of care

This theme relates to GPs’ personal encounters working with people with knee pain attributed to a DMT, focused on their experience of relational aspects of care. In the main, GPs worked to maintain relationships with patients while simultaneously trying to balance patient priorities with their own beliefs about the best course of action. In some cases, GPs described how they stalled or slowed down clinical decisions in an attempt to delay any escalation of care. This theme also describes the challenging emotional experiences of GPs working with this group, including feeling ‘stuck’, having exhausted conservative management options and the difficulty of caring for patients within a health system under strain.

GPs described feeling pressure to arrange injections, imaging or referral to orthopaedics in response to patient expectations. This was particularly the case regarding imaging, where GPs often perceived the patient expected a scan or x-ray, and this expectation was at odds with their own beliefs about best practice. GPs typically described either accommodating patient preferences, or negotiating with the patient to find a compromise:

*“So maybe we make a deal of, let’s give this two weeks … and that we’ll make the decision together as to whether we need to step into the next frame of intervention” (GP14)*.

Explaining or justifying their decision-making to patients was a time and energy consuming process for some GPs. Where patients had a strong preference for a particular intervention i.e. injection or imaging, several GPs described how it was easier to acquiesce to these demands than trying to “*swim against the tide*” when a patient was insistent:

*“It’s a great way to end a consultation to say you’ll order a scan. If you’re not going to do an MRI, you have to do an awful lot of explaining. Often, it’s just the easiest way” (GP4)*.

Another reason given for conceding to patient demands was to maintain the doctor-patient relationship. The potential to “*break the patient relationship”* (*GP9)* when a patient’s expectations were not met was acknowledged, with one GP explaining *“I may not see them for a long time again” (GP12)*.

Some GPs had perceived a shift in the dynamics of the doctor-patient relationship away from GP controlling decision making towards care that was more led by patient views and expectations:

*“I mean, they’ve gone from, “oh you’re the doctor, whatever you say is grand” to “I want this, I want that, I want the other” (GP3)*.

This feeling GPs had of care dictated by the patient was also exemplified by the practice of patients requesting referrals for imaging or orthopaedics over the phone, being reluctant to attend in-person. This left GPs feeling excluded from a care pathway dictated by the patient or physiotherapist *“they want the MRI … then they want the referral for the orthopaedic surgeon, and I am simply a barrier or an enabler … I am essentially an arranger of referrals” (GP5)*:

Another important aspect of the GP-patient interaction was reassuring patients about the nature of their knee condition. Particularly, more experienced GPs emphasised their role in allaying patient concerns by examining the knee and setting positive patient expectations because *“if given time these things will probably sort themselves out” (GP6).* A key component of this approach was about slowing down the decision-making process to allow natural history to take its course:

*“I say “Well do you want to try the exercise for maybe three months first and see how you get on and often there is a peak in the pain and the natural history is, it will be a lot better in 3 months anyway, do you want to wait?” (GP11)*.

Patients were generally amenable to this approach when GPs explained it was about delaying rather than withholding further interventions or imaging. An established and trusting therapeutic relationship was viewed as an advantage in these conversations, particularly when it involved delaying any escalation of care:

*“When they trust you, you have the option of “Look we will see how this goes for a few weeks and come back in a couple of weeks and we will review it again”. You know they are much more amenable to that” (GP1)*.

GPs expressed a strong sense of solidarity and empathy with their patients, particularly for those who had a prolonged wait for orthopaedic services, while continuing to experience significant knee symptoms. GPs described feeling “*very stuck sometimes”* and that their “*hands are tied”* with patients who had not improved despite exhausting conservative management options. Feelings of helplessness, guilt or unease were expressed by GPs when they felt they had no option apart from prescribing increasing doses of analgesics:

*“Even though you feel so sorry for this patient, and you know it’s wrong pumping them with all this stuff (analgesia), but you know you don’t have anything else to offer” (GP13)*.

Some GPs described feeling isolated, left alone to care for patients and compensate for the failings of the wider health service. GPs also expressed a tension between trying to conserve healthcare resources in a system under strain, while also considering the needs of individual patients:

*“We’re expected to balance being the gatekeepers of resources and having the patient satisfied leaving, and somewhere in between practice medicine in the best we can” (GP2)*.

### Theme 2: GP beliefs about what constitutes best care for patients with DMT

This theme relates to GPs beliefs about what constitutes best care for patients with a DMT. Exercise was considered the first line treatment, but GPs also believed patients often sought a ‘quick fix’, affecting their engagement with exercise. Imaging wasn’t viewed as a necessary part of management by most participants, however some reported that arthroscopy could be beneficial.

GPs believed exercise to be the core treatment approach and the “*the bedrock”* of management. GPs’ preference was for strengthening exercise to reduce stress on the joint. General physical activity was also promoted as part of recovery, with a preference for aerobic exercise “*with reduced weight bearing and impact” (GP11)*:

*“The emphasis for me, is to keep them active. I think if I can keep them active a lot of things will resolve, you know” (GP8)*.

GPs felt that exercise was best prescribed by a physiotherapist and cited a lack of confidence, or not having “*the time, knowledge or expertise*” (*GP13)* to prescribe exercise.

Participants believed that the patient should be proactively involved in treatment and didn’t advocate for the use of passive therapies:

*“You have to get them onboard from day one. You won’t get everybody on board. In fact, you’ll get a very small number of people on board. Most people will want to have the passive stuff done” (GP6)*.

Patients’ lack of engagement was believed to be an important reason for failure to improve with exercise. Adherence to prescribed exercise was seen as the patient’s responsibility, and poor adherence was viewed as a lack of commitment or discipline on their behalf. Linked to this, GPs were aware of high rates of non-attendance at public physiotherapy appointments:

*“And then after they’re coming back and say “oh it’s not getting better” but do they actually do the proper exercise you know to strengthen the muscles and do everything? I’m not quite sure” (GP9)*.

One GP spoke about his efforts to address this lack of engagement by exploring barriers to exercise and judging patients’ readiness to engage:

*“I’ll give them options; explore why they’re not interested. If they’re just not interested, I won’t spend excess time and lecture them and drain myself” (GP2)*.

GPs identified patients’ desire for a “*quick fix*” or “*to have the passive stuff done” (GP4)* such as surgery or an injection as a frequent barrier to engaging patients in a more active recovery:

*“I do think there’s some bit of reluctance for people to go for physio sometimes. It’s the society we live in now. It’s much easier if there are quick fixes to these things. They want a pill for everything in some ways” (GP12)*.

A contraindicatory view was voiced by one older GP who believed nowadays patients had a better understanding of their own role in recovery compared to previous generations:

*“There was a lot more magical thinking then. Now they accept that they’ve to work on it and understand that they have to chip in with the treatment” (GP16)*

While generally surgery was not advocated as first line treatment, some GPs believed arthroscopy was beneficial for patients who didn’t respond to exercise or conservative management and had a large or “*significant tear*” on magnetic resonance imaging (MRI). A subset believed a DMT was a structure that could be sutured or repaired. More commonly participants perceived a meniscal tear in middle-aged adults as part of an osteoarthritic spectrum of “*wear and tear”*. Several GPs intuitively believed that for long-term joint health it might be better to avoid arthroscopy. Contrasting beliefs were expressed about the outcome from arthroscopy, while some thought it led to good outcomes, others felt it increased risks of ‘*arthritic change’ (GP14).*

Most GPs didn’t believe an MRI was necessary and they emphasised this to patients:

*“You’ll be saying, “It probably won’t change your management” I bring it back to that. That just because we have the MRI, it’s not going to change anything, it probably doesn’t have a role” (GP1)*.

They stressed an MRI was not “*a cure*” and some GPs were wary of unearthing findings of questionable significance, that they believed could be distressing for patients:

*“Unfortunately, then it (MRI report) comes back with all this noise around it, which says that things aren’t fine. Then the patient is very distressed when you’re explaining about the bones and osteophytes and things like that going on” (GP4)*.

Several GPs held a contrasting view, believing that an MRI could be reassuring for patients and had value in providing patients with the certainty they wanted. They believed “*a clear diagnosis*” according to MRI could create greater engagement with conservative management and reassurance around decisions not to pursue surgical options:

*“Sometimes it does put it to bed a little bit in their heads that “Okay, I have the MRI now and they’re still saying they’re not going to do any surgery on my knee” (GP12)*.

### Theme 3: How GP practice is enacted within the current healthcare setting

This theme describes how GPs enact their practice within the healthcare setting. Limited access to public physiotherapy and orthopaedic services hampered GPs’ efforts to provide care and negatively impacted patients. GPs sometimes practiced pre-emptive referral to orthopaedics as a response to long waiting times. Referring patients to the orthopaedic surgeon without the expectation of surgery was another practice described by GPs. Patients’ having private health insurance had a key influence on clinical decision making. GPs expressed a need for more musculoskeletal training and patient resources that were directly applicable to the primary care setting.

While GPs believed exercise prescribed by a physiotherapist to be the desirable management approach, access to publicly funded physiotherapy was restricted due to long waiting times:

*“So I say, “Let’s try physio first”. The problem however with the physio is that with the public service, the waiting list at the moment, I think it’s four to six months” (GP11)*.

GPs described a community physiotherapy service that was overloaded and difficult to reach leading to GP’s feeling pressure to prescribe more pain medication or escalate care by referring for further imaging or orthopaedic opinion in response to patient frustration and patient suffering:

*“You refer in and nothing happens. Then, of course, the patient is in pain, so they’re taking pain relief. Then the anti-inflammatories blow their stomach, so they’re on PPIs. Then because they blew their stomach, they’re now put on opioids” (GP13)*

Long waiting times to access a public orthopaedic appointment frustrated most GPs and were a significant barrier to caring for patients with persistent symptoms. Some participants described the futility of trying to influence these waiting times and described the negative impact on patients who “*just deteriorate in front of my eyes” (GP13)* while waiting:

*“It’s a fantasy. It’s fairy-tale. You’ll always refer, but you refer in the absolute knowledge that they’re never going to be seen. No matter how many letters you write you are just bouncing your head off the wall” (GP4)*.

In response to public orthopaedic waiting lists, many, but not all, GPs described the practice of “*early referra*l” to compensate for long waiting times. This practice of pre-emptive referral was in the best interests of the patient, but some GPs acknowledged it resulted in unnecessary referrals:

*“When you refer them, you probably refer them earlier, even if they mightn’t need to be referred. In your own head, because you know they’re going to be years waiting, you don’t want to waste time” (GP17)*.

Several GPs explained that when they referred for an orthopaedic opinion, they understood that a surgical intervention was unlikely but hoped the patient would receive *“good authoritative advice” (GP6)* on the importance of exercise in their rehabilitation. Nonetheless the tendency for patients to “*lean back a bit” (GP16)* was noted whereby patients disengaged from conservative management upon referral to an orthopaedic surgeon:

*“People sometimes say, “Well, there’s nothing else that will fix this or address this until I’m seen in that clinic” and you say, “But things can change over time, and we’re going to talk to the physio” and they would sometimes say “but why do I need to do that? You’ve told me it’s bad enough that I need to go and see an orthopaedic surgeon?”” (GP10)*.

Private health insurance was another key influence on how GPs enact their practice within the healthcare setting. GPs described distinct pathways from the outset for patients availing of public or private care. Caring for patients with private health insurance was “*more straightforward*” and allowed for more “*realistic”* conversations when planning care:

*“At the end of the consultation I ask him “Look do you have insurance? Do you have you have private cover because we can get this sorted relatively quickly?” and you’re almost dreading the answer if he doesn’t, because like your consultation and your management plan is totally different” (GP10)*.

Private care was not always viewed as a positive influence on clinical decision making. It was perceived by many GPs as a key driver of patient demands for more imaging or escalation of care beyond physiotherapy. Also in the private sector some GPs had observed a trend for arthroscopy to be performed more frequently or more hastily “*it can be jumped into a bit*” *(GP3).* While other GPs contradicted this viewpoint:

*“I think the surgeons treat every knee the same. I think they do really. I wouldn’t think they wouldn’t do something for a public patient that they wouldn’t do for a private” (GP17)*.

Finally, GPs acknowledged that although musculoskeletal care was a core part of their caseload, it was under-represented in their training and ongoing education:

*“I never did an orthopaedic rotation and yet here I am, the nuts and bolts are back pain, knee pain, hip pain” (GP16)*.

There was a desire for more musculoskeletal training that was directly applicable to the primary care setting. GPs also expressed a need for more patient friendly resources for exercise prescription and self-management:

*“I find it hard to find resources online for patients that are consistent … something that is practical, an entry point to low level ‘let’s get started’ pain management and strengthening exercises” (GP3)*.

*“Obviously, if you can access physio, great just go and work with the physio, but if they don’t, having an online tool, “Here’s your web link. You’re going to get a virtual program here through this.” That would be ideal in helping people navigate that” (GP14)*.

## Discussion

### Summary of main findings

This study explored GPs’ experiences of working with people with knee pain attributed to a DMT. We found that GPs endeavoured to maintain relationships with patients while simultaneously trying to balance patient priorities with their own beliefs about treatment (theme 1). GPs generally believed exercise therapy was the preferred first line treatment for a DMT, but there were challenges engaging patients with this approach and patients were frequently referred to secondary care and some for arthroscopy (theme 2). Finally, wider health service factors influenced their practice within the Irish healthcare setting, such as limited or delayed access to publicly funded physiotherapy and orthopaedic services, and the influence of private health care (theme 3). Pre-emptive referral to orthopaedics and prescription of more analgesics were described by some as responses to long waiting times for healthcare services.

### Comparison with existing literature and implications for practice

Relational aspects of care were central to GPs’ experiences of working with people with DMTs. Our findings reflect the array of inter-personal skills GPs employ in caring for patients with this type of knee pain including empathy, judgement of clinical situations, positive communication, building trust and rapport, and collaboration. There is increasing recognition of the importance of these ‘soft’ skills in the management of specific musculoskeletal conditions [24]. A key part of the clinical encounter described by GPs was ascertaining and balancing patients’ expectations and preferences with their own beliefs about best practice. Patient preferences frequently described by GPs included the expectation of imaging or of ‘a quick fix’ or passive treatment. Although involving patient preferences in the decision-making process remains a key facet of shared decision making [25], the patient preferences described by GPs in this study reinforce the continuing gap between public and consumer expectations, and best practice in the management of persistent musculoskeletal conditions [26]. For the necessary paradigm shift in musculoskeletal care to occur a change in the predominant public understanding of the need for a “structural diagnosis and a surgical fix” is essential [26].

Maintaining the therapeutic relationship was also a priority for GPs and an established rapport was seen as key to getting treatment ‘buy-in’ or agreement around delaying any escalation of care. The positive influence of this therapeutic alliance on patient satisfaction, adherence, and treatment efficacy in healthcare is well recognised [27]. GPs sometimes felt pressurised into conceding to strongly held patient preferences for imaging or surgery as a necessary part of maintaining a harmonious relationship. These findings are echoed by low back pain research, whereby managing patient expectations and a desire to avoid conflict in the patient relationship, influenced implementation of evidence-based treatment in general practice [28]. Other health professionals like physiotherapists also described selecting back pain interventions that facilitated a relationship with and satisfied the patient [29].

GPs in this study were strong advocates of exercise and believed it to be the core treatment approach. In previous studies GPs have expressed ambivalence and uncertainty about the effects of exercise and physical activity in chronic hip and knee pain related to osteoarthritis [30], but we did not find that to be the case. GPs perceived that strengthening exercise prescribed by an expert was the optimal approach, and their preference to rely on the expertise of the physiotherapist is echoed by studies with GPs on knee osteoarthritis management [31]. Despite GPs’ beliefs about strengthening exercises, there is no consensus on the best type of exercise for people with meniscal lesions [32]. A range of exercise approaches are acceptable and efficacious for people with a DMT and knee osteoarthritis [32, 33], therefore, an expert knowledge of strengthening exercise specific to the knee should not be a prerequisite to exercise prescription for these patients. This is important given the limited access to physiotherapy services described in this study, particularly for public patients. Nevertheless, most GPs described lacking the confidence or expertise to prescribe exercise for this knee pain population. Lack of training has previously been cited as a barrier to exercise prescription by GPs [34]. Training for GPs should address skills to prescribe exercise aligned with patient’s goals and preferences, and address patient barriers to engagement [34]. GPs participants also requested access to trustworthy online resources that would facilitate their exercise prescription and provide a ‘stop-gap’ while patients waited for a physiotherapy appointment. Web based physical activity intervention, while not investigated specifically in DMTs are found to be effective for people with osteoarthritis [35]. The ability to direct patients to a such an online resource may help reduce the burden on the GP consultation, which may not be exclusively concerned with the knee, while also developing GP skills and confidence to conservatively manage musculoskeletal disorders.

Non-operative care was described as first line treatment for a DMT by GP participants, however GPs sometimes referred patients to the orthopaedic surgeon. The principal reason cited was failure to respond to conservative management. A thorough understanding of what therapies have been tried and failed and the extent of ‘buy in’ on the patient’s behalf is important information to inform GP decision making, before stepping up to more invasive levels of care [36]. In other painful musculoskeletal disorders such as hip arthroscopy [37] and knee arthroplasty [38] patients may not receive optimal non-surgical care before being escalated to surgery. Several GPs spoke about efforts to resist pressure from patients seeking a ‘quick fix’ and referral for a specialist opinion. They described slowing down their decision making thereby allowing time for recovery to take place. Research suggests most people with knee pain attributed to DMTs appear to have a benign natural history, at least in the medium term, but improvements take time to consolidate. One prospective study tracking people with a DMT over 2 years found the majority had improved function irrespective of treatment [39] with even patients classified as ‘early improvers’ taking up to 12 months to return to normal function. This evidence could justify GPs in asking patients to commit to a more extended period of non-operative management, in turn potentially reducing the number of referrals to secondary care. In our study GPs sometimes referred to secondary care to have their conservative management approach re-enforced by the orthopaedic team. Providing GPs with access to a specialist musculoskeletal care by resourcing of allied-health professional led interface clinics could provide timely assessment and reassurance for complex cases and may allay the need to escalate to secondary care. [40].

In this study a small number of GPs sustained a belief in arthroscopic surgery, particularly for large or ‘significant’ meniscal tears despite research clearly demonstrating that DMT partial meniscectomy does not provide meaningful benefits over non-surgical management. In fact, the larger the tear the poorer the outcome from arthroscopy [41], and total meniscectomy can be associated with more rapid acceleration of degenerative changes [42]. Utilisation of MRI in the middle-aged knee in the absence of red flags has been discouraged due to the imprecise relationship between pain and degenerative findings [43, 44]. The majority GP view was that MRI would not alter the conservative care plan and this has been demonstrated in the Irish secondary care setting [45]. International research goes further, reporting that overutilisation of knee MRI in primary care leads to higher health care utilisation without superior health outcomes, [46, 47] and a higher arthroscopy rate in the imaged group [48]. GPs reported the demand for MRI was frequently patient led, particularly amongst those with private health insurance. These finding suggest that the onus cannot rest solely on GPs to reduce unnecessary musculoskeletal imaging, and wider public health messaging is required to address social pressure from patients seeking imaging [16, 49].

### Strengths and limitations

Strengths of the study were the large and purposive sample of GPs interviewed for this qualitative study. Purposive sampling was carried out to include variation in participating GPs and this may strengthen the transferability of the results. Included clinicians had a range of experience, worked in rural and urban areas, and in practices with varying proportions of private and public patients. In addition, the interviewer strove to be reflexive about her influence on data collection and analysis [23], to mitigate against any potential bias relating to clinical background. The inclusion of an occupational therapist, a GP and an orthopaedic surgeon in the research team also helped reduce the influence of this individual researcher’s experiences and views on the interpretation. Member checking was carried out and these participants agreed that the results were reflective of opinions they expressed during interview. In terms of limitations, these findings must be interpreted in light of the self-selected sample of GPs who volunteered for this study, aware that the interviewer was a practising physiotherapist. It’s possible they responded in a socially desirable manner by over-emphasising the role of physiotherapy or exercise therapy in their approach. In addition, issues related to health service access featured prominently in the data. These issues will vary depending on the structures and organisation of the local and national health system, and our results may reflect our specific setting in the South and Mid-West region of Ireland. Nonetheless, issues with accessing services for musculoskeletal disorders management are an internationally recognised problem [50].

## Conclusions

Findings highlight that GP beliefs about what constitutes best care for DMTs are aligned to the evidence base, with a preference for patient engagement with exercise therapy, while generally, surgery was not advocated as first line treatment. Nonetheless, GPs ability to enact these beliefs was influenced by the challenges of maintaining a harmonious clinician-patient relationship and balancing patient preferences, limited access to conservative management options such as physiotherapy and lack of GP confidence or resources to prescribe exercise. Future efforts to improve management of patients with this type of knee pain necessitates addressing barriers at a service level such as resourcing of primary care physiotherapy services and improving GP confidence and skills to manage this presentation.

## Data Availability

The dataset for this study is not publicly available due to stipulations of ethical approval but can be made available from the corresponding author upon reasonable request.

## List of Abbreviations

DMT: Degenerative meniscal tear
MRI: Magnetic resonance imaging
